# Consequences of cross‐season demographic correlations for population viability

**DOI:** 10.1002/ece3.10312

**Published:** 2023-07-12

**Authors:** Kate Layton‐Matthews, Tone K. Reiertsen, Kjell‐Einar Erikstad, Tycho Anker‐Nilssen, Francis Daunt, Sarah Wanless, Robert T. Barrett, Mark A. Newell, Mike P. Harris

**Affiliations:** ^1^ Norwegian Institute for Nature Research FRAM Centre Tromsø Norway; ^2^ Centre for Biodiversity Dynamics CBD Norwegian University of Science and Technology Trondheim Norway; ^3^ Norwegian Institute for Nature Research Trondheim Norway; ^4^ UK Centre for Ecology & Hydrology, Bush Estate Penicuik UK; ^5^ Department of Natural Sciences Tromsø University Museum Tromsø Norway

**Keywords:** Atlantic puffin, demographic correlations, integrated population model, multi‐population studies, population dynamics, seabird, transient‐LTRE

## Abstract

Demographic correlations are pervasive in wildlife populations and can represent important secondary drivers of population growth. Empirical evidence suggests that correlations are in general positive for long‐lived species, but little is known about the degree of variation among spatially segregated populations of the same species in relation to environmental conditions. We assessed the relative importance of two cross‐season correlations in survival and productivity, for three Atlantic puffin (*Fratercula arctica*) populations with contrasting population trajectories and non‐overlapping year‐round distributions. The two correlations reflected either a relationship between adult survival prior to breeding on productivity, or a relationship between productivity and adult survival the subsequent year. Demographic rates and their correlations were estimated with an integrated population model, and their respective contributions to variation in population growth were calculated using a transient‐life table response experiment. For all three populations, demographic correlations were positive at both time lags, although their strength differed. Given the different year‐round distributions of these populations, this variation in the strength population‐level demographic correlations points to environmental conditions as an important driver of demographic variation through life‐history constraints. Consequently, the contributions of variances and correlations in demographic rates to population growth rates differed among puffin populations, which has implications for—particularly small—populations' viability under environmental change as positive correlations tend to reduce the stochastic population growth rate.

## Graphical abstract

Demographic correlations are pervasive in wildlife populations and can represent important secondary drivers of population growth. We compared the relative importance of survival‐reproduction correlations for population growth rates in three widely geographically separated Atlantic puffin populations. Demographic correlations were positive for all three populations, but their strength differed, likely as a result of differing foraging conditions.

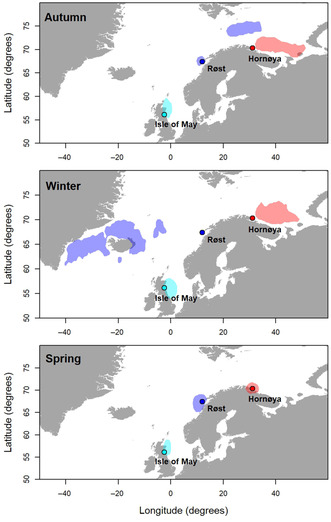

## 1 INTRODUCTION

Temporal correlations in demographic rates appear to be pervasive in wildlife populations (Koons et al., 2009; Morris et al., 2006). This has led to a growing theoretical (Davison et al., 2013; Doak et al., 2005) and empirical (Childs et al., 2011; Fay et al., 2020; Jongejans et al., 2010) evidence base for the importance of covariation in demographic rates as secondary drivers of population growth rates, in addition to direct contributions from demographic rate variances (i.e., primary drivers), particularly in the light of current climate change.

Changing environmental conditions can lead to temporal correlations in population‐level demographic rates. Positive correlations can occur when multiple demographic rates respond positively to an environmental factor or, alternatively, when environmental factors themselves are temporally or spatially correlated (e.g., Jenouvrier et al., 2015). Similarly, negative correlations can reflect independent, contrasting responses of vital rates to the same environmental variable (e.g., Knops et al., 2007) or trade‐offs between demographic rates in response to the same external factor, for example, increased reproduction under favourable conditions can lead to increased mortality (Jongejans & De Kroon, 2005). Empirical evidence suggests that positive correlations among demographic rates are more common than negative correlations, emphasising the importance of shared effects of environmental stochasticity on multiple demographic rates, for example, good conditions generate years with high demographic rates (Ezard et al., 2006; Fay et al., 2020). For long‐lived, iteroparous species, although variability in survival of mature individuals should be limited (Gaillard & Yoccoz, 2003), there is potential for temporal correlations in survival and productivity to be generated in fluctuating environments over their lifetimes (Sutherland et al., 1986).

A study by Fay et al. (2022) found that a species' life history as indicated by their generation time, was a poor predictor of temporal correlations in demographic rates, but rather that environmental factors were generally the primary driver. The importance of environmental stochasticity as a driver of demographic correlations is supported by the findings from several studies (Compagnoni et al., 2016; Fay et al., 2020; Knops et al., 2007). Therefore, the direction and/or magnitude of population‐level demographic correlations and, thus, their contribution to annual population growth should be a function of the environmental conditions a population experiences and, therefore, vary in space.

Population‐level demographic correlations can be cross‐seasonal, either because of carry‐over effects of environmental conditions across seasons, acting via individual condition, or environmental processes may themselves be correlated. Carry‐over effects occur when environmental conditions in one season affect the subsequent condition of individuals and, in turn, their demographic rates, with implications for population dynamics (Harrison et al., 2011; Inger et al., 2010; Paniw et al., 2019). For example, high food availability in the non‐breeding season can improve individuals' survival and body condition, and thus improve breeding success in the succeeding breeding season (Robinson et al., 2020). Survival outside the breeding season relates to physiological processes (e.g., migration, Fayet et al., 2017) and environmental conditions (e.g., food availability, Kautz et al., 2020), which influence individuals' mortality. A positive population‐level correlation between adult survival (year *t* − 1 to *t*) prior to the breeding season, that is, ‘pre‐breeding survival’, and productivity (year *t*), can reflect how environmental conditions during the non‐breeding season affect processes during the breeding season (e.g., Inger et al., 2010; Milner et al., 2013; Veiberg et al., 2017). Conversely, a correlation between productivity (year *t*) and adult survival the year after breeding (year *t* to *t* + 1) can reflect carry‐over effects of environmental conditions in the breeding season (e.g., food availability, Fischer, 2007) on survival in the subsequent non‐breeding season (Cruz‐Flores et al., 2021). However, to our knowledge, the relative importance of cross‐season correlations, reflecting the influence of non‐breeding versus breeding season conditions, has not been explored in a multi‐population context. Such cross‐season correlations should be particularly relevant for populations, for example, migratory populations, affected by environmental factors occurring over a large spatial scales (Both et al., 2006).

Population‐level co‐fluctuations in demographic rates, due to seasonal correlations in environmental stochasticity (or shared demographic responses), have strong implications for long‐term population viability (e.g., Compagnoni et al., 2016; Maldonado‐Chaparro et al., 2018). All else being equal, negative correlations reduce the variance in population growth (Jongejans et al., 2010; Maldonado‐Chaparro et al., 2018), while positive correlations increase annual fluctuations in population size. Increased population size fluctuations reduce long‐term population growth (Compagnoni et al., 2016; Tuljapurkar & Orzack, 1980), which can increase extinction risk, particularly in small populations (Boyce et al., 2006; Tuljapurkar & Orzack, 1980). However, despite their potential importance as drivers of population size fluctuations, correlations in demographic rates are rarely considered in population viability analyses, which can lead to overoptimistic population forecasts (Doak et al., 2005).

**FIGURE 1:**
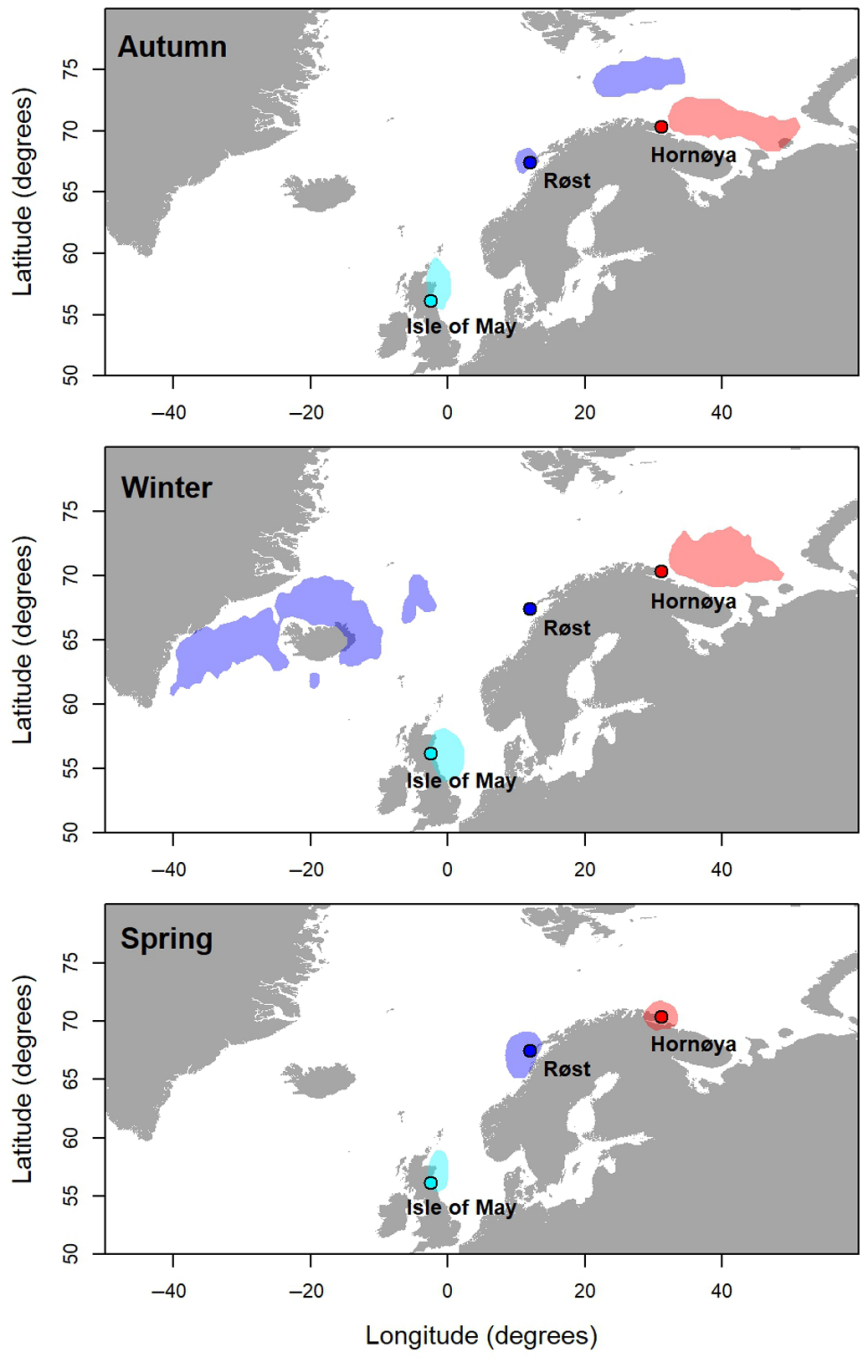
Core non‐breeding distribution (50% kernel utilisation distribution) of Atlantic puffins from; Isle of May (turquoise), Røst (blue) and Hornøya (red), during Autumn (August–September), winter (December–January) and spring (April).

Here, we model temporal variances and covariances using long‐term demographic data from three well studied Atlantic puffin (*Fratercula arctica*) populations (Harris et al., 2005; Harris & Wanless, 2011). Seabirds such as puffins are classical long‐lived species and generally exhibit high and stable adult survival with lower and more variable productivity (Erikstad et al., 2009). They have a prolonged period of immaturity and do not recruit to breeding populations until they are several years old (Bird et al., 2020). Many seabird populations are undergoing drastic, widespread declines and seabirds are one of the most threatened bird groups globally (Dias et al., 2019; Lees et al., 2022; Paleczny et al., 2015). Seabirds are considered highly sensitive to environmental change, particularly through bottom‐up effects from fluctuations in prey resources (Cairns, 1988). Each puffin population in this study experiences different environmental conditions throughout their annual migratory cycles, in three different sea areas of the North Atlantic (Figure 1). For each population, we ran two versions of an integrated population model (IPM): one where pre‐breeding adult survival (year *t* − 1 to *t*) was correlated with and subsequent productivity (year *t*) and the second where productivity (year *t*) was correlated with subsequent adult survival (i.e., post‐breeding survival from year *t* to *t* + 1). We compared cross‐season correlations in adult survival and productivity to determine the extent to which their magnitude and direction differ as a result of environmental conditions. Furthermore, we quantified the contribution of variances in adult survival and productivity and their covariance to variation in population growth (i.e., population viability) using a transient‐life table response experiment (transient‐LTRE).

## 2 METHODS

### 2.1 Study systems

We use long‐term data from three populations of a long‐lived seabird, the Atlantic puffin, with known non‐breeding distributions, to determine if temporal correlations in survival and productivity differ among geographically separated populations. Atlantic puffins (hereafter ‘puffin’) breed in colonies on coastal islands or cliffs. They have delayed maturity (age of first breeding typically 5–7 years). Females lay a single egg in a burrow or under boulders and both males and females take part in incubation and chick rearing (Harris & Wanless, 2011). Here, we studied three puffin populations breeding far apart in the North Atlantic: Isle of May National Nature Reserve (56°11′ N, 2°34′ W), Røst (67°26′ N, 11°52′ E) and Hornøya (70°23′ N, 31°09′ E). To confirm the geographic separation of the three populations also in the non‐breeding season, distributions of the three populations were derived from tracking data using light‐level loggers (geolocator sensors, GLS) deployed between 2014 and 2019 (see Reiertsen et al., 2021 for further methodological details). Across seasons and years, a total of 20,203, 27,752 and 14,898 non‐breeding positions from 149, 204 and 133 individuals were retrieved from Isle of May, Røst and Hornøya, respectively, from which 50% kernel utilisation distribution (UD) were produced (Figure 1). Birds from the three colonies did not overlap in their core (50% UD) foraging areas during either the breeding season or non‐breeding season. Migratory distances also differed greatly, with birds from Røst migrating furthest compared to Hornøya and Isle of May (Figure 1). Individuals from Isle of May vary in their non‐breeding distributions, where studies indicate that approximately half the population remains in the North Sea while the other make excursions into the east Atlantic (Harris et al., 2013), but these are mostly of relatively short durations and hence do not show up in the 50% kernel UD (Figure 1). Puffins from Hornøya are distributed in the south‐east of the Barents Sea and stay there during the whole non‐breeding season, while Røst puffins are located in the central Barents Sea in autumn but migrate to spend the winter in the ocean‐areas south‐east of Greenland and north of Iceland (Figure 1).

### 2.2 Demographic data

Mark‐recapture histories of adult puffins were available for 699 individuals at Isle of May (1984–2019), 572 individuals at Røst (1990–2019), and 952 individuals at Hornøya (1990–2019). Breeding puffins were caught and marked with individually coded colour‐rings or a unique colour ring combination. Birds were captured either in the nest burrow (Isle of May, Hornøya), with noose traps (Hornøya) or in mist nets erected on the colony surface (Røst). Visual resighting of ringed birds was conducted in subsequent years, predominantly in the areas where puffins had been ringed. Productivity data consisted of annual numbers of fledged chicks (F_*t*_) from a sample of monitored pairs (E_*t*_) that made a breeding attempt (defined as egg laid on Isle of May and Hornøya and egg hatched on Røst). Island‐wide population counts (C_*t*_) of adult breeding pairs were conducted at each colony. For Røst and Hornøya, the number of breeding pairs (based on the number of apparently occupied burrows) were upscaled from counts in study plots made every year during the study period (1990–2019; see Anker‐Nilssen & Røstad, 1993 for methodological details for Røst). At the Isle of May, total population counts of occupied burrows were made in 1984, 1989, 1992, 1998, 2003, 2008, 2013 and 2017.

### 2.3 Integrated population model

Precise estimation of temporal demographic correlations can be challenging and requires incorporation of multiple sources of uncertainty. We parameterised an integrated population model (IPM) for each puffin population to jointly analyse adult capture‐mark‐resight (CMR) and productivity data and counts of breeding pairs, following Lahoz‐Monfort et al. (2017). IPMs jointly model multiple demographic timeseries accounting for imperfect detection (Schaub & Abadi, 2011), while allowing for the estimation of parameters where data are not directly available (Abadi et al., 2017).

#### 2.3.1 Estimation of adult survival and productivity

Adult CMR histories were modelled as m‐arrays with a Cormack‐Jolly‐Seber (CJS) model. Annual adult survival rates (Φ_ad,*t*_) were modelled on the logit scale with a Bernoulli distribution, logit(Φ_ad,*t*_) = μ_Φ_ + ε_Φ,*t*_, where μ_Φ_ is the intercept and ε_Φ,*t*_ is a year random effect. Annual resighting rates (*p*_*t*_) were modelled as time dependent, with a one‐year trap‐dependence structure to distinguish between individuals resighted the previous year and those that were not, with constant α (Grosbois et al., 2009), logit(p_*t*_) = μ_*p*_ + ε_*p*,*t*_. We assumed no difference in survival and resighting rates between sexes (Harris & Wanless, 2011). The coefficient α was 2.34 (95% credible intervals: 2.12, 2.57) for Isle of May, 1.65 (1.4, 1.89) for Røst and 2.15 (2.01, 2.29) for Hornøya, that is, individuals were more likely to be resighted the year after capture. Estimates of annual resighting rates, with and without the additive effect of trap‐dependence, are shown in Appendix S1. Productivity data (number of fledged chicks, J_*t*_, from a number of adult pairs, E_*t*_) were modelled as a binomial process J_*t*_ ∼ bin (E_*t*_, F_*t*_), where F_*t*_ is the productivity at each colony in year *t*, logit(F_*t*_) = μ_F_ + ε_F,*t*_.

#### 2.3.2 Estimating temporal demographic correlations

The population model was formulated based on two temporal correlations for adult survival and productivity since productivity is measured during the breeding season but survival spans two consecutive years, from summer in year *t* to summer in year *t* + 1. For the first (‘pre‐breeding’ survival and productivity), adult survival (Φ_ad,*t*_) in year *t* − 1 to *t* was correlated with productivity in year *t*. In the second formulation (productivity and ‘post‐breeding’ survival), productivity (*F*_*t*_) in year *t* was correlated with adult survival in year *t* to *t* + 1. We modelled temporal variation and covariation in Φ_ad,*t*_ and *F*_*t*_ by assuming their temporal random effects (ε_*t*_) followed a multivariate normal distribution on the logit scale, that is, their temporal variances arose from a random process with zero mean but demographic rate‐specific deviations. Temporal random effects were assumed to be shared among individuals and so are at the population‐, rather than individual‐, level. The variance–covariance matrix was modelled using a Cholesky decomposition with parameter expansion, and employs normal conjugate priors, following Chen and Dunson (2003). Details of the modelling of the variance–covariance, and a coded example, are found in Fay et al. (2022) (Appendix S2). We calculated the correlation between adult survival and productivity (*r*) by dividing the covariance by the sum of their temporal variances.

#### 2.3.3 Population count model

A state‐space model was used to link observed population counts (C_*t*_) to the true population size (N_*t*_), assuming an observation error (de Valpine & Hastings, 2002). The system process describes the true population size *N* at year *t* as a function of the previous year's population size (N_*t*−1_). The observation error was assumed to be normally distributed *y*_*t*_ ∼ Normal (N_*t*_, ${\sigma^{2}}_{y}$). Counts for Isle of May were only available for 8 years and so to initialise the population model, which requires estimates of *N* for years 1 to *d*, imputed values were drawn from a normal distribution with mean and standard deviation based on counts in the nearest two census years to mimic natural fluctuations in population size (Zhao et al., 2019).

We only considered the female component of the population and, therefore, model the number of breeding pairs. The total number of breeding females (*N*_*t*_) at year *t* is the sum of the number of new female recruits (*R*_*t*_) and the number of surviving breeding adults (S_*t*_). *R*_*t*_ was modelled as binomial process, assuming age at first breeding (*d*) to be 5 years old, and where breeding females (*N*_*t*−*d*_) in year *t* − *d* produce a single egg, which has the probability *F*_*t*−*d*_ to hatch and the chick survive to fledging, which was multiplied by 0.5 to account for an equal sex ratio at fledging. Finally, we did not have data to directly estimate age‐specific immature survival and hence we modelled a constant parameter, Φ_im_, combining survival since fledging to the year before recruitment to the breeding colony, natal philopatry, and immigration into and emigration from the colony (Lahoz‐Monfort et al., 2017). We assumed that survival over the winter prior to recruitment is equal to that of adult birds (Φ_ad,*t*−1_) and so *R* was modelled as

(1) $$R_{t}∼\text{Binomial}\;\left(N_{t-d},F_{t-d}\;\Phi_{\mathrm{im}}\;\Phi_{\mathrm{ad},t-1}\;0.5\right).$$

The number of surviving adults at year *t* (S_*t*_), was also generated by a binomial process, where the rate parameter is adult survival (Φ_ad,*t*−1_).

(2) $$S_{t}∼\text{Binomial}\;\left(N_{t-1},\Phi_{\mathrm{ad},t-1}\right).$$

Although empirical data suggest that a small proportion of individuals with previous breeding experience do not breed each year, we assumed breeding propensity to be equal to one. A sensitivity analysis showed that a lower breeding propensity (0.9), had little effect on estimate of population sizes or demographic correlations (see Appendix S3). We also assumed no dispersal of breeding adults due evidence that once puffins have recruited to breeding colonies they exhibit very high within‐colony fidelity and total fidelity to the colony (Harris & Wanless, 2011).

#### 2.3.4 Model fitting

Posterior distributions of parameters were obtained using Markov Chain Monte Carlo (MCMC) simulations implemented in jags (Plummer, 2012) via the R package ‘jagsUI’ (Kellner et al., 2019). Three chains of 500,000 iterations were run of which the first 50,000 were discarded and every 10th iteration removed. Convergence was assessed by monitoring the trace or trajectories of the posteriors of variances and estimated parameters using the Gelmin‐Rubin convergence statistic $\hat{R}$ for each stochastic node as modified by Brooks and Gelman (1998). Parameter estimates are summarised as posterior means with 95% credible intervals.

### 2.4 Transient‐life table response experiment (LTRE)

We performed a transient‐LTRE to assess the relative contributions from temporal variation and covariation in time‐varying demographic rates (adult survival and productivity) to the variation in realised population growth rates (λ_*t*_), following Koons et al. (2016, 2017). For each cross‐season correlation (i.e., the two formulations of the population model), we decomposed the variance in the realised population growth rate at year *t*, using 10,000 samples from the posterior distributions of demographic rates estimated in the IPM. We used a random design LTRE to decompose the variance in the realised population growth rate, var(λ_*t*_), into contributions from variances and covariances in lower‐level demographic rates and population size:

(3) $${{\text{Contribution}}_{\theta }}^{\mathrm{var}\left(\lambda_{t}\right)}\approx \sum_{j}\mathrm{cov}\left(\theta_{i,t},\theta_{j,t}\right)\;\frac{\partial \lambda_{t}}{\partial \theta_{i,t}}\frac{\partial \lambda_{t}}{{\partial \theta }_{j,t}}.$$

θ_*t*_ is a vector of demographic rates and population structures. Using these sensitivities and covariances among all elements of θ, we obtained a first‐order approximation of the variance in *λ*_*t*_, where sensitivities were calculated at the means of the simulated vital rates. Contributions from variances in demographic rates was given on the diagonal of θ and the covariances on the sub‐diagonal of θ. To facilitate comparison, relative contributions of each term were summarised as scaled contributions by dividing the (co)variance contribution from a given term by the total variance in *λ*_*t*_.

## 3 RESULTS

### 3.1 Demographic rates and breeding adult numbers

**FIGURE 2:**
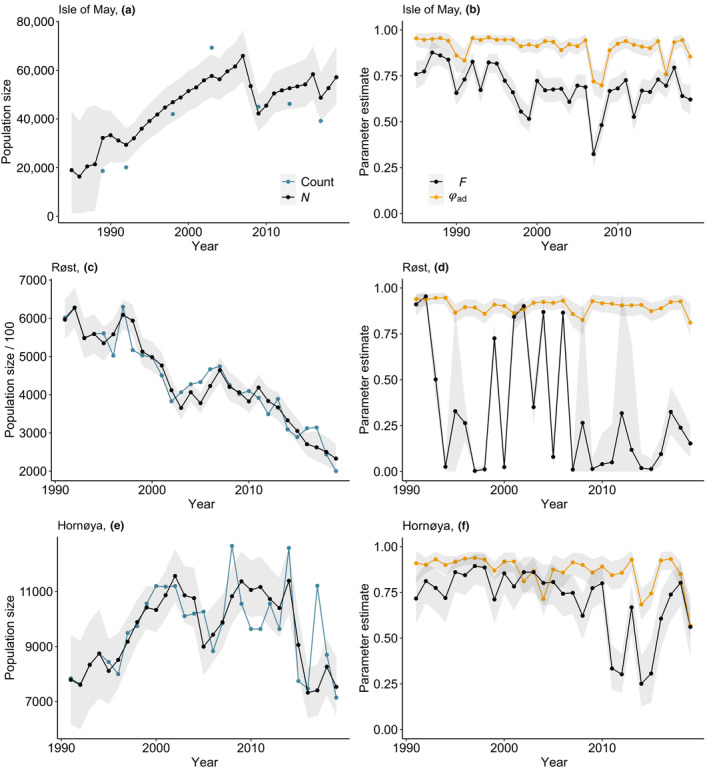
Estimated number of breeding pairs (black) with 95% credible intervals (grey shading) and counts (blue) and posterior means with 95% credible intervals of annual adult survival (orange) and productivity (black) for the populations (a–b) Isle of May, (c–d) Røst and (e–f) Hornøya, based on the pre‐breeding survival (ϕ_ad_,_*t*−1 → *t*_ → F_*t*_) formulation of the IPM.

The geometric mean annual population growth rate was positive for Isle of May ($\bar{\lambda }$ = 1.04; 95% credible intervals = 1.02, 1.09), negative for Røst (0.91; 0.88, 0.93), and stable for Hornøya (0.99; 0.97, 1.00). Based on a linear regression (linear model with a continuous, fixed year effect), there was no temporal trend in population growth rates for Isle of May (slope = −0.003; 95% credible intervals = −0.008, 0.001), Hornøya (−0.003; −0.007, 0.000) or Røst (−0.045, −0.209, 0.120). Initially, breeding numbers at Isle of May increased rapidly, then declined before remaining relatively stable (Figure 2a), while the population at Røst underwent a constant, rapid decline (Figure 2c). Numbers at Hornøya also increased initially, then stabilised before declining gradually in the most recent years (Figure 2e).

Adult survival was high and relatively stable for all populations (Figure 2b,d,f). Mean (95% credible intervals) annual adult survival was 0.92 (0.90, 0.94) for Isle of May, 0.91 (0.89, 0.93) for Røst and 0.89 (0.85, 0.91) for Hornøya. In contrast, mean productivity was high for Isle of May (0.71; 0.66, 0.75) and Hornøya (0.74; 0.66, 0.81) but low for Røst (0.14; 0.04, 0.38). Combined immature survival from fledging until the year prior to recruitment (assumed to be 4 years old) and natal philopatry (Φ_im_) for the Isle of May population was 0.49 (0.41, 0.63), that is, an annual rate of 0.84 (0.780, 0.89). For the Røst population, Φ_im_ was 0.39 (0.32, 0.47) or an annual rate of 0.79 (0.75, 0.83) and for Hornøya, 0.41 (0.37, 0.46) or an annual rate of 0.80 (0.78, 0.82).

### 3.2 Demographic correlations

**FIGURE 3:**
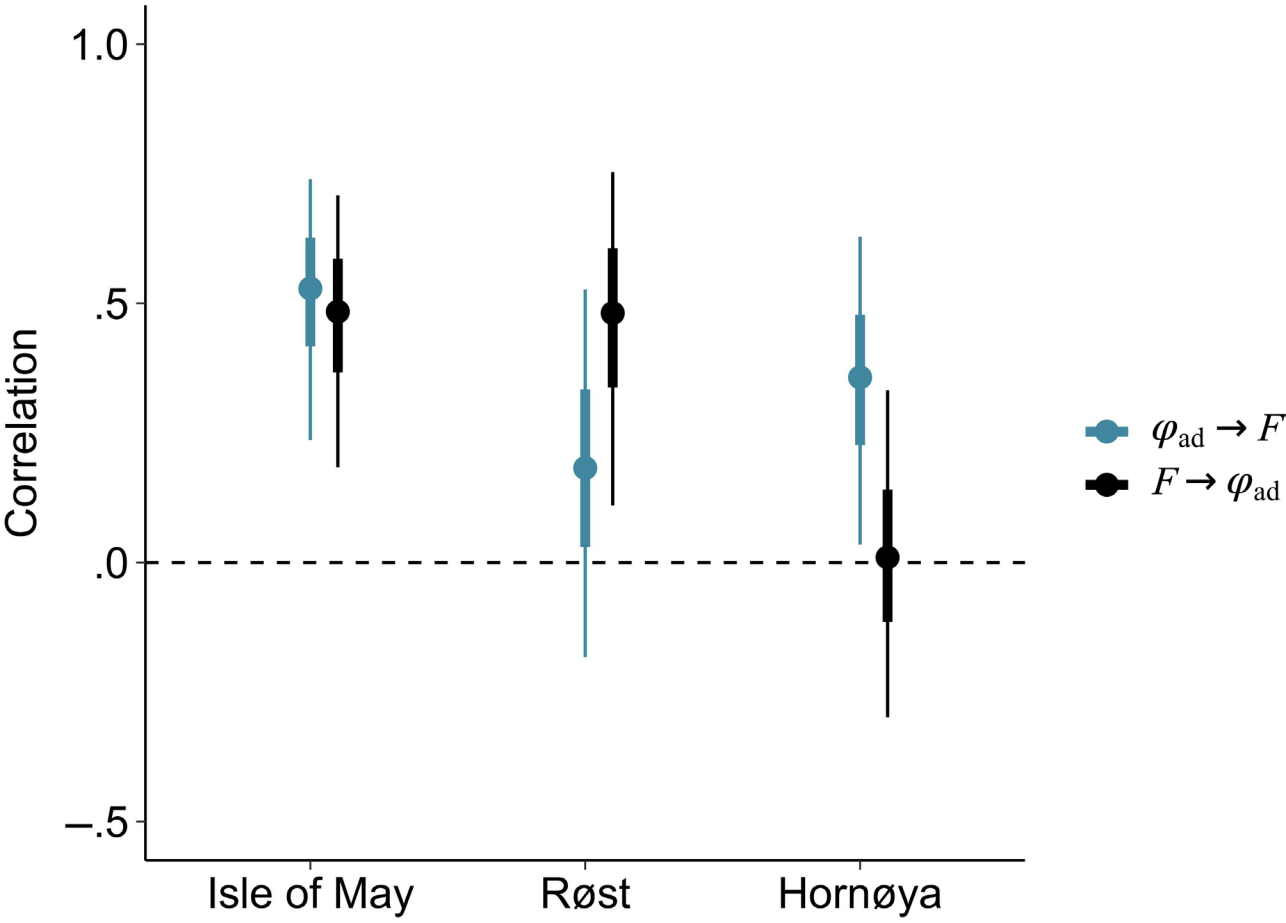
Correlation (mean and 95% credible intervals) between survival prior to the breeding season (*t*−1 → *t*) and productivity at year *t* (ϕ_ad_,_*t*−1 → *t*_ → F_*t*_, in blue) and the correlation between productivity (*t*) and adult survival to the subsequent breeding season (*t* → *t* + 1) (F_*t*_ → ϕ_ad_,_*t* → *t*+1_, black) for populations; Isle of May, Røst and Hornøya.

Temporal correlations between adult survival and productivity were positive in all three populations and for both cross‐season correlations (Figure 3), presented as means (95% credible intervals). For Isle of May, the positive correlation between pre‐breeding survival and productivity (ϕ_ad,*t*−1→*t*_ → F_*t*_) was 0.51 (0.24, 0.74), which was marginally stronger than the correlation between productivity and post‐breeding survival (F_*t*_ → ϕ_ad_,_*t*→*t*+1_ = 0.47; 0.18, 0.71). Both correlations for Isle of May had a 100% probability of being greater than zero (i.e., 95% of the posterior distribution was above zero). For Røst, there was limited statistical support for the correlation (*r*) between pre‐breeding adult survival and subsequent productivity (ϕ_ad,*t*−1→*t*_ → F_*t*_ = 0.18; −0.18, 0.53), but strong support (Pr(*r*) > 0 = 0.98) for the correlation between productivity and post‐breeding adult survival (F_*t*_ → ϕ_ad_,_*t*→*t*+1_ = 0.46; 0.11, 0.75). Conversely, for Hornøya, there was a statistically significant correlation between pre‐breeding survival and productivity (ϕ_ad_,_*t*−1→*t*_ → F_*t*_), equal to 0.35 (0.03, 0.63), but a weak correlation between productivity and post‐breeding survival (F_*t*_ → ϕ_ad_,_*t*→*t*+1_ = 0.01; −0.30, 0.33), with a low posterior probability (Pr(*r*) > 0 = .52).

### 3.3 Contributions to realised population growth rates

**FIGURE 4:**
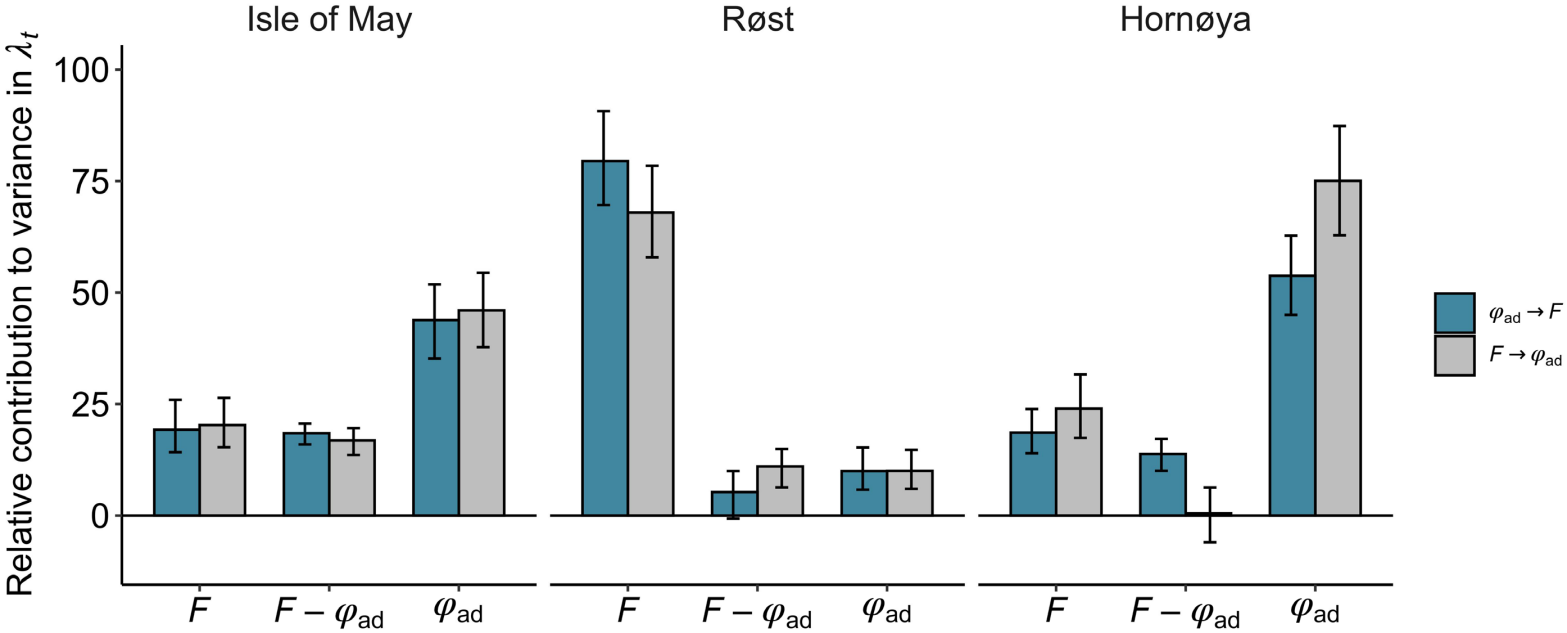
Percentage contributions of variation and covariation in adult survival and productivity to variance in the population growth rate (λ_*t*_) for populations; Isle of May, Røst and Hornøya. The variance decomposition was performed for two formulations of the IPM, that is, for both cross‐season correlations where pre‐breeding survival and productivity (ϕ_ad_,_*t*−1 → *t*_ → F_*t*_) are temporally correlated (blue), versus where productivity and post‐breeding survival (F_*t*_ → ϕ_ad_,_*t* → *t*+1_) are correlated (grey). Bars reflect the mean contribution based on samples from the posterior distributions of demographic rates, error bars represent 95% credible intervals.

The variance in realised population growth rates (λ_*t*_) was decomposed into contributions arising from variation in adult survival, productivity and their temporal covariation, using a transient‐LTRE based on posterior samples from the IPM (Figure 4). Contributions are shown as proportions of the total variance explained. For Isle of May, variance and covariance contributions were similar for both cross‐season correlations, with variance in adult survival explaining around 45% compared to 20% for productivity. The correlation between pre‐breeding survival and productivity explained 16% of the variance in population growth and the correlation between productivity and post‐breeding survival explained 14% (Figure 4). For Røst, for both cross‐season correlations, productivity contributed more to the variation in population growth than adult survival (Figure 4). When productivity was correlated with post‐breeding adult survival (F_*t*_ → ϕ_ad_,_*t* → *t*+1_), this correlation explained 11% of the variation in population growth, productivity explained 68% and adult survival 10%. When adult survival prior to breeding was correlated with productivity (ϕ_ad_,_*t*−1 → *t*_ → F_*t*_), the correlation explained only 5% and credible intervals overlapped zero, while productivity explained 80% and adult survival 10%. For Hornøya, adult survival contributed more than productivity to variance in *λ*_*t*_ in both model formulations (Figure 4). When productivity was correlated with adult survival after the breeding season (F_*t*_ → ϕ_ad_,_*t* → *t*+1_), this correlation contributed little to variance in λ_*t*_ (<1%), while the covariance between ϕ_ad_,_*t*−1 → *t*_ → F_*t*_ explained to 14% of the variance in population growth (Figure 4).

## 4 DISCUSSION

### 4.1 Temporal demographic correlations are positive

Correlations between adult survival and productivity were positive (or overlapping zero) across study populations, for both cross‐season correlations. This supports existing empirical evidence from other species that temporal demographic correlations are most commonly positive, as a result of shared effects of environmental conditions among demographic rates, for example, Sæther and Engen (2002). There is limited empirical evidence of negative correlations between key demographic rates in long‐lived species (but see e.g., Cruz‐Flores et al., 2021; Maldonado‐Chaparro et al., 2018). This may be because any trade‐offs are masked by strong positive correlations induced by environmental variation in long‐lived species (Capdevila et al., 2022). In our study, the three puffin populations used different foraging areas throughout their annual cycle (Figure 1). In winter, individuals migrated to different ocean regions: North Sea (Isle of May), Iceland/Irminger Sea (Røst) and Barents Sea (Hornøya), but also in the breeding season when foraging concentrated in area around the colonies, hundreds of km apart (Figure 1). The diets of the three puffin populations differ during the breeding season and, likely, also during the non‐breeding season. The difference in the strength of demographic correlations among populations, therefore, indicates that environmental conditions, for example, prey resources, are key in generating cross‐season correlations. Besides negative relationships between reproductive effort and survival (e.g., Cruz‐Flores et al., 2021; Erikstad et al., 1998), empirical studies of population‐level correlations in seabirds are lacking (but see Fay et al., 2022). Our results, therefore, provide insight into the relative importance of cross‐season correlations, representing different seasonal processes, as secondary demographic drivers of population growth. Such correlations have been shown in other species, for example, in a population of kestrels (*Falco tinnunculus*), where productivity, largely driven by vole abundance, was positively correlated with juvenile and adult survival (Fay et al., 2020). By increasing population size variability and reducing long‐term growth rates, positive correlations can increase populations' vulnerability to environmental changes. Quantifying these correlations is necessary to perform accurate population viability analyses (Davison et al., 2013) and, thus, is particularly important for threatened species as is the case for the majority of seabirds including puffins (BirdLife International, 2018; Lees et al., 2022).

### 4.2 Environmental conditions as a driver of temporal correlations

In seabirds, inter‐annual fluctuations in food availability represent a likely cause of cross‐season correlations, since conditions affecting prey availability are considered the main drivers of survival (e.g., Reiertsen et al., 2014; Sandvik et al., 2005), productivity (Becker et al., 2007; Cohen et al., 2014) and, therefore, population growth rates (Jenouvrier et al., 2018). While the mechanism(s) behind correlations remains undetermined, positive correlations may reflect independent demographic rate‐responses to the same environmental factor, where multiple seasonal processes within a year can be positively correlated. Environmental correlations are likely stronger when breeding and non‐breeding areas are closer, as a result of spatial scaling of environmental factors (Lande et al., 1999), for example, affecting shared prey resources (Olin et al., 2020). However, relationships with demographic processes can be disrupted by, for example, extreme events (Frederiksen et al., 2008). Positive correlations driven purely by environmental factors have been observed in several taxa, for example in meerkats (*Suricata suricatta*), were high recruitment of dominant breeding individuals, led to reduced emigration of ‘helper individuals’ in the same year, positively affecting population growth (Conquet et al., 2022). The same mechanisms can also explain negative correlations, for example in oak trees where growth and reproduction were both dependent on the same environmental conditions, but in opposite ways (Knops et al., 2007).

### 4.3 Pre‐breeding adult survival and productivity

Environmental conditions in the non‐breeding season can impact productivity in the subsequent season through carry‐over effects on individuals’ body conditions (Betini et al., 2013; Harrison et al., 2011; Inger et al., 2010). Winter is a critical period in the non‐breeding season, particularly at high latitudes, when light and food resources are more limited and climate conditions were often more extreme (Genovart et al., 2013). Winter carry‐over effects on reproduction have been observed in several migratory (e.g., Rockwell et al., 2012) and non‐migratory species (e.g., Veiberg et al., 2017). Furthermore, energy intensive processes such as feather moult occur during this period, where moulting periods are associated with higher mortality (Anker‐Nilssen et al., 2017; Barta et al., 2006; Morales et al., 2007). The mechanism behind demographic correlations for migrant species differs from that of non‐migrants, as migrants are exposed to a wider range of environments during the year, where environmental factors may operate at larger spatial scales (Schaub et al., 2012). We found support for a positive correlation in pre‐breeding adult survival and subsequent productivity for two study populations (Isle of May and Hornøya). Unfavourable conditions with limited food availability in the non‐breeding season can cause higher adult mortality and lower reproductive outcomes, for example, reduced transfer of individual fat deposits across seasons. A positive correlation may, therefore, reflect that winter foraging conditions, a critical period in the non‐breeding season with the harshest conditions for seabird survival (Bogdanova et al., 2011) also affect subsequent reproduction.

Although neither mechanism can be discounted, puffins from Isle of May and Hornøya winter relatively close to their breeding colonies (Figure 1), pointing to an increased likelihood of cross‐season correlations in environmental factors as the underlying cause of strong demographic correlations between pre‐breeding survival and subsequent productivity. Puffins from the Isle of May feed mainly on small pelagic fish in the breeding and non‐breeding seasons and fluctuations in sandeel abundance have been correlated with both adult survival (Harris et al., 2005) and productivity (Frederiksen et al., 2006). The availability of Barents Sea capelin influences productivity and egg investment of puffins breeding at Hornøya (Barrett & Krasnov al., 1996; Barrett et al., 2012). Furthermore, the Barents Sea capelin stock migrate from northern and central Barents Sea towards the Norwegian coast in spring to spawn (Gjøsæter, 1998) and so puffins breeding at Hornøya are likely dependent on capelin as a food source in both breeding and non‐breeding seasons. Climate conditions affect prey distributions and abundances, and thereby indirectly influence seabird survival and reproductive rates, at large spatial scales (e.g., effects of North Atlantic Oscillation on survival, Sandvik et al., 2005).

For the Røst population, there was a lack of support for a correlation between pre‐breeding survival and productivity. Individuals breeding at Røst winter farther from the breeding grounds compared to puffins from the Isle of May and Hornøya (Figure 1), potentially explaining the lack of correlation between pre‐breeding survival and productivity. Alternatively, consistently low productivity, and thus recruitment, due to poor breeding conditions could have led to a ‘decoupling’ of demographic rates at Røst. Such decoupling has been observed in other species, for example, between juvenile survival and reproduction in long‐tailed skuas (*Stercorarius longicaudus*), reflecting an adaption to a strongly fluctuating prey source (Barraquand et al., 2014). Both possible mechanisms can, therefore, represent a form of demographic buffering, that is, a mechanism by which population size fluctuations are buffered against environmental stochasticity (Hilde et al., 2020; Tuljapurkar et al., 2009). Similarly, senescence rates for puffins at Røst were also found to be lower than at Isle of May and Hornøya (Landsem et al., 2023), potentially providing another buffering mechanism that limits population‐level consequences of low and/or variable productivity, via individual trade‐offs.

### 4.4 Productivity and post‐breeding adult survival

Generally, cross‐season correlations between reproduction and post‐breeding survival appear to be uncommon. Variation in survival in long‐lived species is generally buffered against environmental stochasticity, with individuals adopting strategies to reduce costs of current reproduction on future survival and reproduction, for example by skipping breeding or reducing clutch and/or brood size (Gaillard & Yoccoz, 2003). However, poor breeding conditions have been shown to affect post‐breeding survival (Davis et al., 2005; Nichols et al., 1982). For instance, unfavourable breeding conditions in alpine swifts (*Tachymarptis melba*), a long‐distance migrating species, had knock‐on effects on the survival of breeding adults (Robinson et al., 2020). In the case of puffin populations on Isle of May and Røst, there was support for a positive correlation between productivity and post‐breeding adult survival, indicating that favourable breeding conditions were associated with higher adult survival after the breeding season. Consequently, by affecting both productivity and adult survival, any changes in breeding conditions at these colonies could have a big impact on population growth rates. In contrast, there was little support for a correlation between productivity and post‐breeding survival for adults from Hornøya. After the breeding season, adult puffins from Hornøya migrate to an area in the southern Barents Sea identified as a hotspot for many seabird species and potentially with good and predictable foraging conditions (Barrett & Krasnov, 1996; Sandvik et al., 2016). This potentially enables puffins to compensate for any negative effects of poor breeding conditions.

Here, we explored cross‐season correlations between two key demographic rates, adult survival and reproduction. Variability in immature survival, and associated recruitment rates, can contribute non‐negligibly to variation in population growth in long‐lived species, through large inter‐annual fluctuations (Ezard et al., 2006; Reid et al., 2004). However, due to delayed maturity in seabirds, with immatures spending most of the time away from the colony, the importance of immature survival for seabird population dynamics remains an important knowledge gap. Combined immature survival and natal philopatry, which reflects both the proportion of immatures returning to the colony and immigration of puffins from other colonies, was similar to adult survival for Isle of May birds. A study of the same population also found similar immature and adult survival rates (Harris, 1983). Since survival of younger age classes is expected to be lower (and more variable) in seabirds (e.g., Fay et al., 2015; Frederiksen et al., 2008), this high estimate could reflect a combination of a lower immature survival rate and net immigration, since these separate processes cannot be distinguished here. Estimated combined immature survival and natal philopatry was also similar to adult survival for Hornøya birds, but immature and adult survival rates were lower than that found in a previous study (Sandvik et al., 2008).

## 5 CONCLUSION

By comparing cross‐season correlations across three geographically distinct populations, our study provides empirical evidence for the relative importance of cross‐season demographic correlations and how they are likely driven by the environmental conditions that puffins experience throughout their annual cycle. Our findings thus highlight the role of ecological context in understanding a population's dynamics, as the contributions of both variances and covariances in adult survival and reproduction to population growth appear population specific. Such cross‐season correlations providing insights into the main drivers of population change and may be particularly important, and complex, in migratory species experiencing a wider variety of environmental conditions during the year. As positive demographic correlations increase population variability, and thereby a population's extinction risk, understanding the role of temporal covariance is especially important for threatened species. Effects of environmental change can be amplified by positive correlations between demographic rates, making populations with strong correlations more vulnerable. However, weaker correlations may themselves be a response to poorer conditions, where decoupling of demographic rates limits population variance. A better understanding of these cross‐season correlations in conservation studies, and the underlying mechanisms behind them, will contribute to improved knowledge of population responses to environmental change, improved predictions of population viability, and thereby, potentially, more effective conservation.

## AUTHOR CONTRIBUTIONS

**Kate Layton‐Matthews:** Conceptualization (equal); formal analysis (lead); methodology (lead); validation (lead); visualization (lead); writing – original draft (lead); writing – review and editing (equal). **Tone K. Reiertsen:** Conceptualization (lead); data curation (equal); project administration (equal); writing – review and editing (equal). **Kjell‐Einar Erikstad:** Conceptualization (equal); data curation (equal); funding acquisition (lead); writing – review and editing (equal). **Tycho Anker‐Nilssen:** Data curation (equal); writing – review and editing (equal). **Francis Daunt:** Data curation (equal); writing – review and editing (equal). **Sarah Wanless:** Data curation (equal); writing – review and editing (equal). **Robert T. Barrett:** Data curation (equal). **Mark A. Newell:** Data curation (equal); writing – review and editing (supporting). **Mike P. Harris:** Data curation (equal); writing – review and editing (equal).

### OPEN RESEARCH BADGES

This article has earned an Open Data badge for making publicly available the digitally‐shareable data necessary to reproduce the reported results. The data is available at [https://github.com/katel‐m/Puffin‐single‐IPMs].

## Supporting information


Appendix S1.
Appendix S2.
Appendix S3.
Appendix S4.

## Data Availability

Data supporting the results are archived in Dryad data repository: https://doi.org/10.5061/dryad.1zcrjdfz1.
