# Supplementary material for: Consequences of cross‐season demographic correlations for population viability
Source: Ecol Evol. 2023 Jul 12;13(7):e10312. doi: 10.1002/ece3.10312 (PMC10338798; doi:10.1002/ece3.10312)
Supplement: Supplementary file 1 — Appendix S1. Appendix S2. Appendix S3. Appendix S4. [file ECE3-13-e10312-s001.docx]

**Supplementary Information for ‘Consequences of cross-season demographic correlations for population viability’. Layton-Matthews *et al.***

**Appendix S1.** Annual recapture probabilities estimated using the IPM for (a) Isle of May, (b) Røst and (c) Hornøya.

**
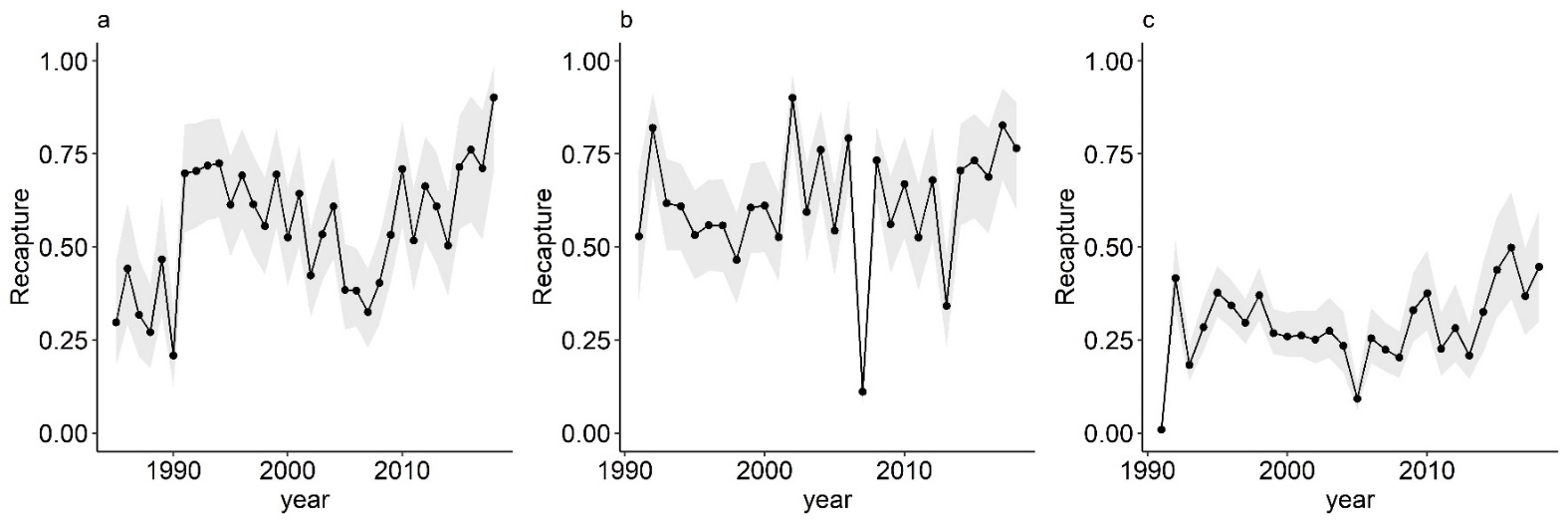
**

**Fig. S1** Annual recapture probability estimates (for individuals not caught previous year, to calculate recapture probability α coefficient should be added to each annual estimate) for (a) Isle of May, (b) Røst and (c) Hornøya.


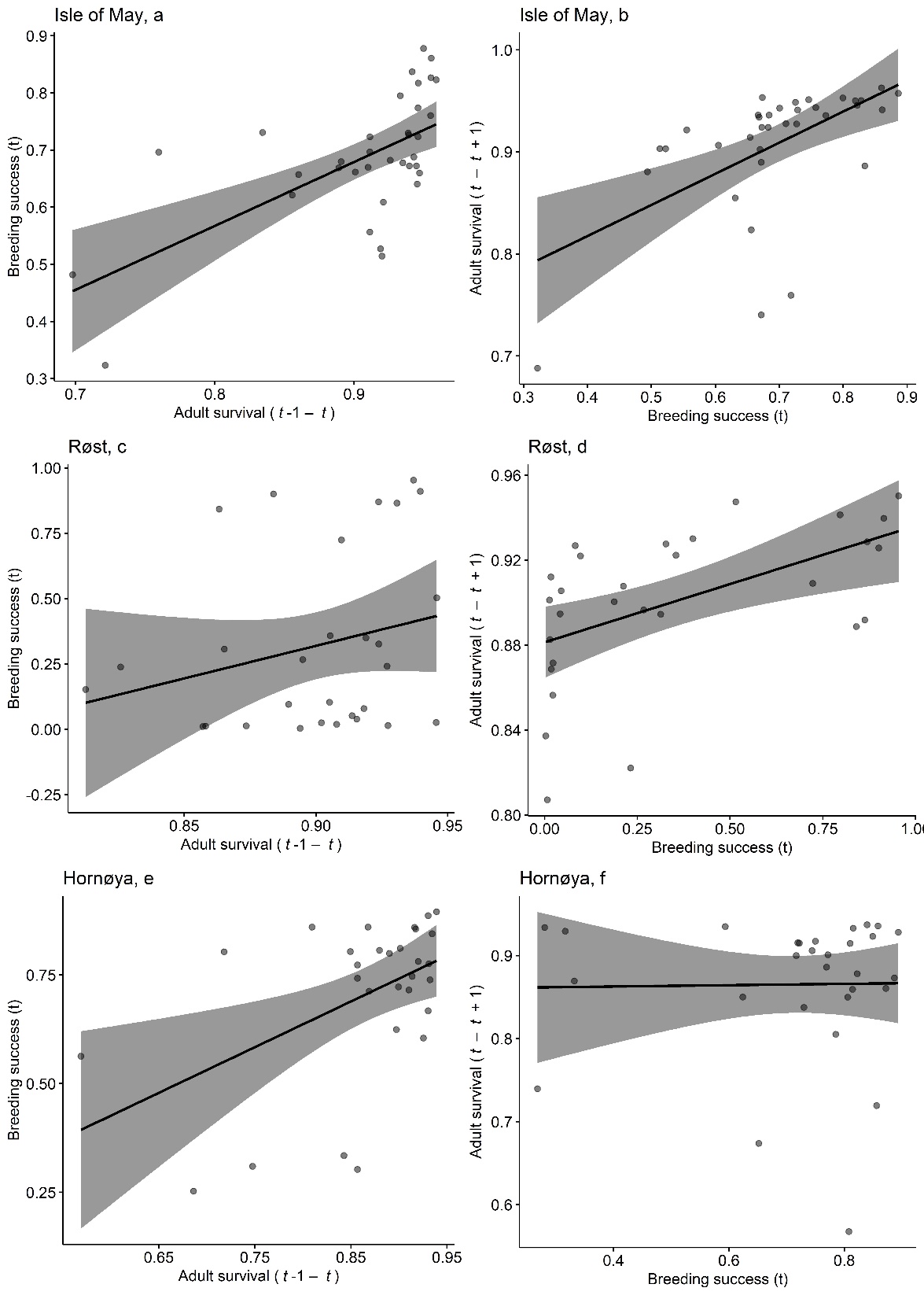
**Appendix S2** Temporal correlations in adult survival and breeding success.

**Fig S2.** Annual estimates of adult survival (*t*-1 → *t*) against annual breeding success (t) (**a, c, e**) and breeding success (t) against adult survival (t – t+1) (**b, d, f**) for Isle of May (**a-b**), Røst (**c-d**) and Hornøya (**e-f**) populations. Regressions slopes were estimated using a linear model with 95% confidence intervals

**Appendix S3** Sensitive analysis of the impact of breeding propensity on population dynamics

As a result of Atlantic puffin life-history characteristics, we know little about the degree to which mature adult puffins (after they have return to their breeding colony) skip breeding, i.e., do not lay an egg. To explore whether a breeding propensity of less than 1 could have potential consequence of that the estimation of demographic parameters and population sizes and therefore the survival-productivity correlation, we re-ran the IPM include a constant parameter (BP) of 0.90, i.e., we assume that each year, 10% of mature puffins do not breed. Therefore, the recruitment model becomes:

R_t_ ∼ Binomial(N*_t_*_−_*_d_*, BP F*_t_*_−_*_d_* Φ_im_ Φ_ad,_*_t_*_-1_ 0.5), where BP = 0.9.

Inclusions of a breeding propensity constant of 0.9 had little effect on population sizes estimations (**Figure 1**) and did not notable affect survival-productivity correlations. Including BP in the models did not alter the posterior distributions of the survival-productivity correlations (means and 95% credible were identical).

**
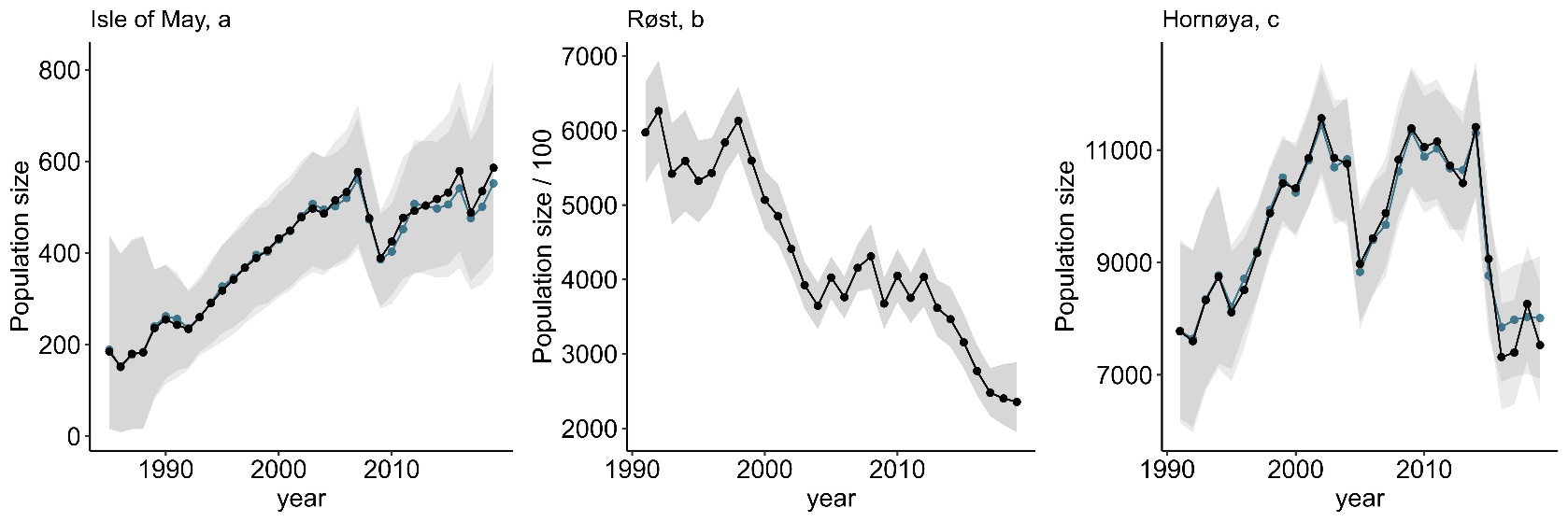
**

**Figure 1.** Annual estimates of population sizes (N*_t_*), for Isle of May, Røst and Hornøya, based on an IPM with the BP constant (90% breeding propensity) included and without it (i.e., the same as in the manuscript).

**Appendix S4** Robustness of estimates of temporal demographic correlations in an IPM framework

As counts were not available for several years for Isle of May colony, this has the potential to bias parameter estimates, and therefore, estimations of demographic correlations. We therefore compared estimates of temporal survival-productivity correlations, for each population, estimated either using the IPM framework, or outside of the IPM framework (using a joint model of survival and productivity via correlation temporal random effects but with linking them to the count data through the state process equation).

**Table 1.** Estimates of survival-productivity correlations using both approaches. Correlations are presented as posterior means with associated 95% credible intervals.

| **Colony** | **Approach** | **cor(ϕ_ad_ → F_t_)** | **cor(F_t_ → ϕ_ad_)** |
| --- | --- | --- | --- |
| Isle of May | IPM (as in manuscript) | 0.51 [0.23, 0.74] | 0.48 [0.20, 0.72] |
|  | No IPM/counts | 0.51 [0.18, 0.77] | 0.47 [0.13, 0.74] |
| Røst | IPM (as in manuscript) | 0.19 [-0.17, 0.54] | 0.49 [0.15, 0.76] |
|  | No IPM/counts | 0.12 [-0.33, 0.55] | 0.41 [-0.04, 0.74] |
| Hornøya | IPM (as in manuscript) | 0.34 [0.03, 0.63] | 0.02 [-0.29, 0.33] |
|  | No IPM/counts | 0.39 [0.00, 0.69] | 0.05 [-0.32, 0.42] |
